# Effectiveness of using different sources of zinc on the productive performance of laying hens

**DOI:** 10.1038/s41598-026-41183-1

**Published:** 2026-03-19

**Authors:** Fayza M. Salem

**Affiliations:** Animal and Poultry Nutrition Department, Desert Research Center, Cairo, Egypt

**Keywords:** Laying hens, Nano-zinc oxide, Zinc carbonate, Zinc oxide, Biochemistry, Physiology, Zoology

## Abstract

Heat stress is a major challenge in the poultry industry, adversely affecting birds’ physiological activity, appetite, growth rate, egg production, and egg quality. This study aimed to mitigate the negative effects of high temperatures on laying hen productivity, egg quality, egg zinc content, specific blood parameters, and nutrient utilization during the summer. The experiment involved feeding different zinc sources to laying hens aged 40 to 54 weeks. Eighty Bovans Brown laying hens were randomly assigned to four groups; each had 10 replicates with two hens per replicate. The treatments included a control group (Con), zinc oxide at 500 mg/kg feed (ZnO), zinc carbonate at 500 mg/kg feed (ZnC), and nano-zinc oxide at 50 mg/kg feed (NZn). The zinc experimental treatments resulted in higher egg yield (*P* < 0.05), egg mass, egg weight, serum zinc levels (*P* < 0.01), and feed conversion ratio (*P* < 0.05) compared to the control. When compared to other treatments, nano-zinc oxide produced the lowest AST levels, while zinc carbonate produced the greatest reduction in ALT levels. The zinc oxide group exhibited the greatest serum calcium level. However, no significant impacts were observed in terms of feed intake, nutrient digestibility, egg zinc content, total protein, albumin, globulin, triglycerides, or malondialdehyde. The findings revealed that including zinc carbonate, zinc oxide, and nano-zinc oxide in laying hens’ diets throughout the summer season boosted egg output and egg weight while improving the feed efficiency without compromising the hens’ well-being.

## Introduction

Heat stress is a major challenge in the poultry industry, occurring under high ambient temperature and humidity, and it negatively affects egg production, feed intake, feed efficiency, and survival^1^. Heat stress impairs growth performance, intestinal morphology, digestive and antioxidant enzyme activities, and beneficial gut microbiota, while increasing oxidative stress, feed conversion ratio, and pathogenic bacteria populations^2^. It also elevates stress and inflammatory markers, including corticosterone, D-lactate, IL-1β, and HSP70 expression, while reducing mucosal immune indicators such as secretory IgA and IL-10 in chickens^3^. Zinc is an essential trace element required for normal physiological functions in hens, including growth, muscle development, reproduction, and egg production^4,5^. It is a structural or catalytic component of more than 300 enzymes, including dehydrogenases, anhydrases, polymerases, phosphatases, peptidases, kinases, and deaminases^6^. Zinc also activates carbonic anhydrase, a key enzyme involved in carbonate formation and eggshell mineralization^7^. Zinc supplements are widely used in broiler and layer diets because many feed ingredients are inherently low in bioavailable zinc. In monogastric animals, zinc bioavailability is limited (approximately 6–11%) and is influenced by its chemical form, diet composition, bird age and physiological status, and interactions with other minerals^8^. Zinc deficiency impairs bicarbonate ion secretion, resulting in reduced eggshell quality^9^. Since zinc is not stored in the body, continuous dietary supplementation is required, particularly under hot climatic conditions where zinc demand is increased^10^. Under heat stress conditions, zinc protects cells by enhancing antioxidant capacity, scavenging reactive oxygen species, and modulating heat shock responses. Zinc stimulates metallothionein synthesis, which functions as an effective free-radical scavenger^11^. As a component of key antioxidant enzymes such as glutathione peroxidase, superoxide dismutase, and glutathione S-transferase, zinc helps limit oxidative damage^12^. In addition, zinc interacts with transition metals and antioxidant vitamins (A and E), thereby reducing lipid peroxidation in zinc-supplemented birds^13^. Zinc is commonly incorporated into poultry diets in the form of inorganic sources such as zinc oxide or zinc carbonate, owing to their low cost and commercial availability. Nevertheless, the use of nano-zinc, despite its higher cost, has gained attention as a means to enhance zinc bioavailability compared with conventional sources. Among the metal nanoparticles produced globally, zinc oxide nanoparticles represent the third most abundant type and are distinguished by their uniform size and morphology, extensive surface area, high surface reactivity, superior catalytic efficiency, and strong adsorption capacity^14^. Nanoparticles of zinc oxide are specially engineered mineral salts with particle sizes ranging from 1 to 100 nm^15^. These nanoparticles possess notable antimicrobial and antioxidant properties and have been shown to support reproductive performance in poultry. Advances in nanotechnology have enabled the use of nano-zinc in poultry diets to improve trace mineral utilization, productivity, eggshell quality, antioxidant status, bone development, and selected blood biochemical parameters^16^. Supplementation of nano-zinc oxide (5–25 mg/kg) in laying hen diets has been shown to enhance egg production rate, egg mass, and feed efficiency^17^. Similarly, nano-zinc increased eggshell thickness more effectively than inorganic zinc sources^18^. Consistent with these findings, higher dietary nano-zinc oxide levels (20–60 mg/kg) improved egg weight, egg output, and egg mass while maintaining feed intake^19^. On the other hand, dietary supplementation with zinc hydroxide at levels of 50 and 100 mg/kg feed significantly improved broiler performance, feed intake, villus height, crypt depth, and the villus height-to-crypt depth ratio compared with the control group^20^. Additionally, adding 50 and 100 mg/kg of zinc methionine to broiler diets under heat stress boosted body weight gain, glutathione peroxidase activity, and crude protein utilization while lowering total cholesterol^21^. The National Research Council^22^ states that the ideal zinc amount in poultry diets is 40 mg/kg, independent of source. However, multiple studies have not found the appropriate amount of zinc consumption in laying hen diets, which would affect performance, egg quality, and output. The purpose of this research is to contrast the impacts of a nano-zinc source and two types of inorganic zinc sources (zinc carbonate and zinc oxide) on egg output, egg quality, nutrient digestibility coefficients, some blood profiles, and zinc concentration in inner egg components in laying hens aged from 40 to 54 weeks during the hot season. The study hypothesizes that inorganic zinc sources, especially zinc carbonate and nano-zinc, can mitigate the detrimental effects of heat stress on laying hens’ productivity.

## Results

### Productive performance

Table 1 presents the impacts of various zinc sources on the productivity of chickens during the hot climatic conditions. The treated groups recorded a significant increase in hen-day egg production. (*P* < 0.05) and egg mass (*P* < 0.01) while improving the feed conversion ratio (*P* < 0.05), with the most pronounced effects noticed in the zinc carbonate group, followed by nano-zinc oxide and zinc oxide, in contrast to the untreated group. Additionally, all zinc-supplemented groups showed boosted egg weight relative to the untreated group. However, there were no discernible variations in feed consumption among the treatments.

**Table 1:** Impact of different types of zinc on the productivity of laying hens during the summer season.

Item	Con	ZnO	ZnC	NZn	SE	*P* value
Treatments						
Hen day egg production%	68.0^c^	69.2^bc^	82.2^a^	78.5^ab^	3.27	0.0236
Egg mass (g)	37.7^c^	42.1^b^	48.8^a^	46.8^ab^	1.60	0.0017
Egg Weight (g)	55.6^b^	60.8^a^	59.4^a^	59.6^a^	0.875	0.0062
Feed Intake (g/hen/day)	78.8	74.9	73.4	72.3	5.16	0.822
FCR (Feed/Emass)	2.10^a^	1.80^ab^	1.52^b^	1.55^b^	0.143	0.0486

a, b,c Means within the same row showing different letters are significantly different,.

Con = Control, ZnO = Zinc oxide, ZnC = Zinc carbonate, NZn = Nano-Zinc oxide, SE = Standard error. FCR= Feed conversion ratio, Emass = Egg mass.

### Egg quality characteristics

Table 2 illustrates the impact of various zinc forms on characteristics of egg quality during the hot climate conditions. In comparison to the control, all experimental treatments resulted in a significant increase (*P* < 0.01) in egg weight and shell surface area. The zinc carbonate treatment led to the lowest shell percentage (*P* < 0.05) among the treatments. There were no notable variations found among treatments for albumen percentage, yolk percentage, Haugh unit, or shell thickness.

**Table 2:** Impact of different types of zinc on egg quality traits during the summer season.

Item	Con	ZnO	ZnC	NZn	SE	*P* value
Treatments						
Egg weight (g)	52.7^b^	61.0^a^	62.2^a^	59.4^a^	1.89	0.0032
Albumen%	62.1	60.6	63.0	60.2	2.72	0.889
Yolk%	26.8	25.4	24.3	26.8	1.28	0.429
Haugh unit	90.0	90.8	85.4	91.5	2.94	0.485
Shell%	13.3^a^	12.3^ab^	10.8^b^	12.1^ab^	0.582	0.0354
Shell thickness (mm)	0.471	0.405	0.403	0.450	0.025	0.142
Shell surface area (cm^2^)	65.2^b^	72.3^a^	73.3^a^	70.9^a^	1.59	0.0029

a, b Means within the same row showing different letters are significantly different, Con = Control, ZnO = Zinc oxide, ZnC = Zinc carbonate, NZn = Nano-Zinc oxide, SE = Standard error.

### Digestibility trial

Table 3 shows the impact of various forms of zinc on nutrient digestibility under the hot climate conditions. The digestibility of dry matter, organic matter, crude protein, and ether extract did not significantly differ among the treatments.

**Table 3:** Impact of different types of zinc on digestibility of nutrients during the summer season.

			Treatments			
Item	Con	ZnO	ZnC	NZn	SE	*P* value
Digestibility%						
Dry matter	82.1	81.3	78.1	81.4	2.84	0.780
Organic matter	83.6	79.2	83.0	78.8	2.31	0.358
Crude protein	69.8	63.0	67.9	68.6	3.05	0.399
Ether extract	84.6	81.7	86.6	82.1	2.00	0.343

Con = Control, ZnO = Zinc oxide, ZnC = Zinc carbonate, NZn = Nano-Zinc oxide, SE = Standard error.

### Mineral composition of internal egg components (yolk and albumen)

Table 4 presents the effect of different types of zinc on the mineral composition of internal egg components (yolk and albumen) during the hot conditions. The treated groups had a notable rise (*P* < 0.05) in magnesium (Mg) content, while copper (Cu) and nickel (Ni) levels decreased significantly (*P* < 0.05 and *P* = 0.0001, respectively) in contrast to the untreated group. The lowest manganese (Mn) concentration (*P* < 0.01) was observed with the zinc oxide treatment. Zinc carbonate treatment, followed by zinc oxide caused the lowest aluminum (Al) levels (*P* < 0.01) in opposition to the control. The nano-Zn oxide treatment led to the lowest cobalt (Co) concentration (*P* < 0.05) among all treatments. The amount of chromium (Cr) was greatly lowered (*P* = 0.01) in both the zinc oxide and nano-Zn oxide treatments compared to the others. There were no discernible variations in the zinc (Zn), iron (Fe), calcium (Ca), cadmium (Cd), barium (Ba), silicon (Si), strontium (Sr), and lead (Pb) among the treatments.

**Table 4:** Impact of different types of zinc on mineral content of internal egg components during the summer season.

Item	Con	ZnO	ZnC	NZn	SE	*P* value
Treatments						
Zn (ppm)	55.0	47.8	42.4	50.7	3.13	0.0816
Fe (ppm)	197	231	229	230	12.3	0.229
Mg (ppm)	280^b^	362^a^	348^ab^	404^a^	24.7	0.0272
Ca (ppm)	856	871	928	1015	89.6	0.593
Mn (ppm)	4.53^a^	1.30^b^	5.35^a^	3.39^ab^	0.720	0.0030
Cu (ppm)	38.5^a^	13.4^b^	10.3^b^	7.28^b^	5.63	0.0145
Al (ppm)	34.6^a^	16.3^bc^	9.51^c^	30.1^ab^	4.66	0.0042
Si (ppm)	62.3	32.7	25.7	40.8	8.83	0.0628
Co (ppm)	5.66^ab^	9.42^a^	7.92^a^	3.24^b^	1.46	0.0357
Cd (ppm)	2.16	4.41	4.05	3.75	0.585	0.0606
Cr (ppm)	11.4^ab^	7.04^b^	15.7^a^	9.11^b^	1.79	0.0100
Ba (ppm)	3.40	2.05	3.41	3.66	0.512	0.118
Ni (ppm)	57.7^a^	33.6^b^	27.6^b^	27.3^b^	4.61	0.0001
Sr (ppm)	1.61	1.40	1.53	1.93	0.241	0.462
Pb (ppm)	13.7	19.2	22.6	19.8	3.15	0.347

a, b,c Means within the same row showing different letters are significantly different, Con = Control, ZnO = Zinc oxide, ZnC = Zinc carbonate, NZn = Nano-Zinc oxide, SE = Standard error.

### Blood biochemical profiles

The impact of various sources of zinc on serum biochemical parameters during the summer season were displayed in Table 5. Supplementation with various zinc forms had no discernible impact on albumin, globulin, or total protein, triglycerides, or malondialdehyde concentrations. Zinc supplementation significantly (*P* < 0.001) raised serum cholesterol concentration, with the zinc oxide group showing the greatest levels when contrast to the control. Serum ALT activity was the lowest (*P* < 0.01) in hens added with zinc carbonate, followed by nano-zinc oxide and the control group, whereas the highest activity was detected with zinc oxide supplementation. Similarly, serum AST activity was the lowest (*P* < 0.05) in the nano-zinc oxide group contrast to the others. A significant elevation (*P* < 0.01) in the level of calcium in the blood was noted with zinc oxide supplementation rather than the others. In addition, all zinc-supplemented groups revealed a noteworthy rise in serum zinc concentration (*P* < 0.01), with the highest values recorded in the zinc oxide group in contrast to the control.

**Table 5:** Impact of different types of zinc on some serum parameters during the summer season.

Item			Treatments			
	Con	ZnO	ZnC	NZn	SE	*P* value
Total protein (g/dL)	4.45	5.73	4.26	4.10	0.513	0.135
Albumin (g/dL)	2.14	2.53	1.96	1.85	0.232	0.204
Globulin (g/dL)	2.31	3.20	2.31	2.26	0.450	0.394
Cholesterol (mg/dL)	121^c^	269^a^	168^b^	172^b^	12.8	0.0003
Triglycerides (mg/dL)	352	391	273	209	79.6	0.439
ALT (U/L)	101^b^	115^a^	89.9^c^	90.7^bc^	3.37	0.0015
AST (U/L)	360^a^	346^ab^	340^ab^	323^b^	7.38	0.0432
Malondialdehyde (nmol)	56.2	61.4	44.7	52.3	5.55	0.213
Calcium (mg/dl)	9.47^b^	10.7^a^	9.41^b^	9.43^b^	0.220	0.0046
Zinc (µmol/L)	120^c^	252^a^	185^b^	182^b^	14.5	0.0015

a, b,c Means within the same row showing different letters are significantly different, Con = Control, ZnO = Zinc oxide, ZnC = Zinc carbonate, NZn = Nano-Zinc oxide, SE = Standard error.

## Discussion

Poultry are highly susceptible to heat stress due to the absence of sweat glands and their reliance on panting for thermoregulation. Elevated ambient temperatures negatively impact the health, physiological status, productivity, and efficiency of laying hens^23^. To our knowledge, this study is the first to investigate the beneficial effects of zinc carbonate in laying hens’ diets, particularly during hot season. The current results demonstrated significant improvements in hen-day egg production, egg mass, egg weight, and feed conversion ratio, particularly with zinc carbonate treatment followed by nano-zinc oxide treatment without negative impacts on hens’ health. It is known that when hens pant due to heat stress, they lose excessive carbon dioxide (CO_2_), causing respiratory alkalosis (increased blood pH). This CO_2_ loss disrupts the acid-base balance, reducing available bicarbonate needed for eggshell formation^24^. When zinc carbonate enters the stomach of hens, it interacts with the stomach juice (HCl) to release soluble zinc chloride, water, and CO₂. This CO_2_ compensates for the lost amount of bicarbonate, which is needed for eggshell formation during the hot climate. Additionally, the water produced from this reaction is essential for hens. Additionally, zinc is converted into ionic form, which is necessary for absorption and activates the carbonic anhydrase enzyme^7^. This enzyme converts carbon dioxide into bicarbonate ions. These bicarbonate ions are then transformed into calcium carbonate by the hen, the primary component of the eggshell. Also, the improvements in the productive performance can be ascribed to zinc’s vital role as a cofactor for numerous enzymes involved in the metabolism of proteins, carbohydrates, energy, and nucleic acids^6^. Likewise, nano-zinc may be an appropriate source to replace minerals in the feed. These can be utilized at lower levels and can deliver greater results than standard zinc sources while indirectly preventing environmental contamination^15^. The present results agree with Abou-Ashour et al.^17^ and Fawaz et al.^19^, who reported that dietary nano-zinc oxide supplementation (5–60 mg/kg) significantly improved egg production, egg mass, egg weight, and feed efficiency in laying hens. Similarly, Saleh et al.^21^ observed enhanced performance in broilers fed zinc-supplemented diets under hot conditions. However, Karimi et al.^25^ found no effects of nano-zinc oxide or zinc oxide supplementation on Haugh unit or feed intake in Japanese quail, while Cufadar et al.^26^ reported no significant effects of zinc level or source on laying performance, indicating that zinc responses may vary with species, dosage, source, and environmental conditions. The current study demonstrated that dietary zinc supplementation increased the surface area of eggshells, which may be explained by increased carbonic anhydrase activity in the eggshell gland and plasma, boosting the development of shells^27^. However, zinc supplementation had no discernible effect on other egg and shell quality characteristics. Mineral antagonism could account for this lack of reaction because too much zinc can lower copper bioavailability, which could prevent future increases in egg and shell quality^28^. Similar to our results, adding 40, 80, and 120 mg of nano-ZnO/kg to the feed of Hy-Line laying hens had no impact on the eggs’ quality^18^. The experimental treatments had no beneficial effect on the nutrient digestibility coefficients. This could be due to high ambient temperatures; around 32 °C, the activity of several enzymes, including trypsin, chymotrypsin, and amylase, all drastically decreases^29^. In the small intestine, these vital pancreatic digestive enzymes convert food into nutrients that can be absorbed. The current results are consistent with those of Fawaz et al.^19^, who discovered that adding nano-zinc oxide to laying hens’ diet at levels of 20, 40, and 60 mg Zn/kg had no effect on nutritional digestibility. The mineral composition of eggs and eggshells is influenced by diet composition, rearing system, genetic background, mineral source, and level, and management conditions^30^. Although limited information is available on the effect of dietary zinc on egg mineral composition, the present study showed that zinc supplementation increased magnesium deposition in internal egg components. This may be attributed to the complementary roles of zinc and magnesium in enzyme activity and transcriptional regulation, as well as their synergistic absorption^31^. Consistent with these findings, Yenice et al.^32^ reported that dietary zinc supplementation enhanced the deposition of minerals in eggs of laying hens. The reduced copper deposition in internal egg components observed in this study can be attributed to the antagonistic interaction between zinc and copper, whereby high zinc intake decreases copper availability^33^. Consistently, previous studies have shown that increasing dietary zinc elevates iron concentrations while reducing copper levels in several hen tissues^34^. Additionally, the present study revealed that zinc supplementation decreased the deposition of other metals, including aluminum, silicon, nickel, cobalt, and chromium, likely due to zinc-induced metallothionein synthesis. Metallothioneins are cysteine-rich proteins that bind essential and heavy metals, thereby reducing their bioavailability and limiting their deposition in egg components^11,35^. The present study found no significant differences in zinc content among the experimental treatments. This agrees with Plaimast et al.^36^, who reported no effect of high dietary zinc supplementation (600 mg/kg) on albumen zinc concentration in laying hens. In contrast, Abedini et al.^18^ observed increased yolk zinc levels with nano-zinc oxide supplementation. The discrepancy may be attributed to the fact that the present study analyzed combined albumen and yolk rather than individual egg components. The blood biochemical results showed that dietary zinc supplementation increased serum zinc concentrations in laying hens. This increase is attributed to zinc absorption in the small intestine by passive diffusion, where its transport within enterocytes is regulated by metallothionein, a zinc-binding protein whose synthesis is induced by high dietary zinc intake. Metallothionein binds zinc, preventing its binding to intestinal protein for subsequent transport to the blood^37^. In agreement with these results, Yu et al.^38^ demonstrated that dietary zinc supplementation at 35 or 70 mg/kg markedly increased serum zinc levels in laying hens. Serum ALT and AST are biomarkers of liver damage, and zinc supplementation can reduce their levels by enhancing antioxidant defense, particularly via Cu/Zn-superoxide dismutase, and stabilizing hepatocyte membranes^12^. Several studies have reported reduced serum ALT and AST in broilers fed nano-zinc oxide at various inclusion levels^39,40^. However, Zhang et al.^41^ observed no significant changes in these enzymes with nano-zinc oxide supplementation, suggesting that the hepatic response to zinc may depend on dosage, source, and experimental conditions. The present findings are consistent with Gholizadeh et al.^9^, who reported that zinc supplementation (30 mg ZnO/kg) did not affect serum triglyceride levels under either normal or heat-stress conditions. Similarly, Elsayed et al.^42^ observed an increase in serum cholesterol in Japanese quail fed 100 mg ZnO/kg. In addition, Salim et al.^43^ and Sarvari et al.^44^ reported that neither the dietary level nor the source of zinc significantly affected serum total protein or albumin concentrations. Malondialdehyde (MDA) levels indicate lipid peroxidation. In contrast to the present results, serum MDA levels decreased linearly with increasing dietary supplementation with 30 or 60 mg/kg of zinc sulfate and zinc picolinate^45^. The ways that the present study’s findings differ from those of previous research may be ascribed to variations in zinc type and level, bird species, geographic area, temperature, humidity, and other factors.

## Conclusion

The current study concluded that the addition of zinc carbonate (500 mg/kg diet), zinc oxide (500 mg), and nano-zinc oxide (50 mg) in the diets of laying hens (40–54 weeks old) during the summer months positively helped in improving the productive performance and the efficacy of feed utilization. The most notable improvements were observed with zinc carbonate treatment. Additionally, the data indicated that nano-zinc oxide supplementation had no negative impacts on hens’ health.

## Materials and methods

### Birds’ management and housing

This investigation was completed in Ras-Sedr City (South Sinai Experimental Research Station) despite the scorching weather. This station is owned by the Desert Research Center in Cairo, Egypt. Ras-Sedr has a desert environment, which means that temperatures vary from extremely hot in the summer to pleasant in the cooler months. Eighty Bovans Brown laying hens were randomly assigned to four groups; each had 10 replicates with two hens per replicate. These hens were purchased from a local commercial layer farm. The treatments were Con (control group), ZnO (500 mg zinc oxide/kg feed), ZnC (500 mg zinc carbonate/kg), and NZn (50 mg nano-zinc oxide/kg). The hens were raised in wire cages within triple-deck batteries. An electronic digital thermo-hygrometer was used to measure the indoor ambient temperature and relative humidity. The chickens were exposed to sixteen hours of continuous light while being grown in cages made of wire within triple-deck batteries. The diet formula was displayed in Table 6^22^, which contained 18.5% CP and 2620 Kcal ME/kg. Feed and water were available as needed.

### Manufacture method of nano-zinc oxide

Nano-zinc oxide was synthesized by hydrolyzing and condensing zinc acetate dihydrate with potassium hydroxide in an alcoholic medium at low temperature. The resulting ZnO nanoparticles settled at the bottom, after which the supernatant was removed and the precipitate thoroughly washed with methanol. The obtained precipitate was subsequently dispersed in a methanol–chloroform solution^46^. The synthesized ZnO nanoparticles exhibited a spherical morphology with an average particle size of 30 ± 5 nm.

**Table 6:** The composition of the experimental diet.

Ingredients	%
Yellow corn	57.1
Soybean meal 44%	30.0
Limestone	7.00
Di-Calcium phosphate	2.00
Wheat bran	3.00
DL-Methionine	0.300
Salt	0.300
Vit&Min. Premix*	0.300
Total	100
Calculated Values	
CP %	18.5
ME (Kcal/kg)	2620
Ca %	3.20
AP %	0.490
DL-Methionine%	0.590
L-Lysine % Meth + Cys Zn (mg)	0.970 0.900 174

*Vitamins and minerals premix, each 3 kg include: Vit A 10,000,000 mg, Vit D3 2,000,000 mg, Vit E 10,000 mg, Vit K 1000 mg, Vit B12 10 mg, Vit B1 1000 mg, Vit B2 5000 mg, Vit B6 1500 mg, Pantothenic acid 10,000 mg, Niacin 20 g, Folic acid 1000 mg, Biotin 100 mg, Fe 30,000 mg, Mn 60,000 mg, Choline chloride 600,000 mg, Cu 4000 mg, Zn 50,000 mg, Se 400 mg and Iodine 300 mg.

AP%= Available P%.

### Internal climatic conditions

Table 7 displays the house’s ambient temperature (max, min, average, and THI) and relative humidity (RH%) (max, min, and average). Temperature and humidity were assessed with an electronic digital thermo-hygrometer. The temperature-humidity index (THI) was computed with the following formula^47^:

THI = 0.60Tdb + 0.40Twb.

Tdb represents the maximum temperature of the dry bulb, while Twb represents the minimum temperature of the wet bulb.

**Table 7:** Indoor ambient temperature (TºC), relative humidity (RH %) and temperature humidity index (THI) during the experimental.

Month		T°C				RH%	
	Max	Min	Average	THI	Max	Min	Average
July	37.3	25.6	31.4	32.6	80.3	30.0	55.1
August	37.1	24.6	30.9	32.1	82.1	33.3	57.7
September	35.1	23.1	29.1	30.3	78.3	31.1	54.7
October	31.4	23.4	27.4	28.2	79.2	38.3	58.8
Mean (µ)	35.2	24.2	29.7	30.8	80.0	33.2	56.6

Max = Maximum, Min = Minimum.

### Productive performance and egg measurements

The consumed feed was weekly determined. Egg mass (g/hen/day) was computed as the product of average egg weight and egg number, and feed conversion ratio (FCR; g feed/g egg mass) was obtained by dividing total feed intake by egg mass. When the trial is over, ten eggs per treatment were collected to evaluate egg quality attributes, including egg weight, albumen ratio, yolk ratio, Haugh unit, shell ratio, thickness of the shell, and shell surface area. Albumen, yolk, and shell ratios were expressed as percentages of total egg weight. The Haugh unit was computed by the following Eq. 4^8^:

$$Haugh{\text{ }}unit = {\text{ }}100{\text{ }}\times{\text{ }}log{\text{ }}\left( {Albumen{\text{ }}height + 7.57 - 1.7{\text{ }}\times{\text{ }}Egg{\text{ }}weigh{t^{0.37}}} \right)$$

A micrometer was employed to gauge the shell’s thickness (ST). The formula that follows was used to calculate shell surface area (SA)^49^:

$$SA{\text{ }}\left( {c{m^2}} \right){\text{ }} = {\text{ }}3.9782 \times E{W^{0.7056}}$$

Where: 3.9782 = constant factor, EW = egg weight (g).

### Digestibility trial

During the last three days, fecal specimens were gathered every 24 h from five chickens per group. Excreta and feed intake were recorded, and samples were kept at 65 °C to constant weight and stored for the analysis of dry matter, organic matter, crude protein, and ether extract^50^.

### Mineral composition of internal egg components (yolk and albumen)

The mineral contents of Zn, Fe, Mg, Ca, Mn, Cu, Al, Co, Cd, Cr, Ba, Ni, Si, Sr, and Pb (ppm) in the internal egg components were determined using Inductively Coupled Argon Plasma Spectrometry (ICAP) 6500 Duo, Thermo Scientific, England- 1000 mg/L multi-element certified standard solution, Merck, Germany was considered as stock solution for instrument standardization^51^.

### Blood biochemical profiles

On the trial’s last day, five chickens were randomly chosen from each group to have blood samples drawn from their brachial wing veins (without anesthesia or euthanasia). The method entails safely confining the bird, identifying the vein under the wing, sterilizing the area, and putting a needle into the vein at a shallow angle to collect the specimen. Then, the specimens of blood were immediately centrifuged at 3000 rpm for 20 min, and the serum was kept at −20 °C for further analysis. The following blood parameters were measured: total protein, albumin, cholesterol, triglycerides, alanine transaminase (ALT), aspartate aminotransferase (AST), malondialdehyde, calcium, and zinc. All parameters were determined colorimetrically using “Biodiagnostic kits.” Subtracting albumin from total protein yielded globulin.

### Data analysis

The SAS statistical software was used to analyze the data^52^ by one-way analysis of variance (ANOVA) based on the model that follows: Y_j_ = µ + T_i_ + e_ij_.

Where Y_j_ represents the observation, µ is the overall mean, T_i_ is the fixed effect of treatment (i = 1, 2, 3, and 4), and e_ij_ is the random error term. Differences among treatment means were separated using Duncan’s multiple range test^53^.

## Data Availability

All data generated and analyzed during this study are incorporated into this published article.
